# Ovarian Pregnancy

**DOI:** 10.7759/cureus.31316

**Published:** 2022-11-10

**Authors:** Punit Hans, Gunjan Gunjan

**Affiliations:** 1 Obstetrics and Gynecology, Patna Medical College, Patna, IND

**Keywords:** emergency laparotomy, spiegelberg criteria, ovarian pregnancy, ovarian ectopic pregnancy management, ruptured ectopic pregnancy

## Abstract

Ovarian pregnancy is a rare uncommon presentation of an ectopic pregnancy. Without any known risk factors, ovarian pregnancy seems to appear at random. A 29-year-old female patient with previous two cesarean deliveries visited the gynecology emergency department with the complaint of constant dull aching pain in the lower abdomen, aggravated by postural changes. Per vaginal examination, cervical motion tenderness was present. The patient was admitted, and her transvaginal sonography was done along with a urine pregnancy kit test for the suspicion of ectopic gestation. After the initial treatment and arrangement of two units of packed red blood cells after proper grouping and cross-matching for the patient, laparotomy was done. At the time of surgery, left-sided ruptured ovarian pregnancy was confirmed by the Spiegelberg criteria.

## Introduction

The incidence of ovarian pregnancy ranges from one per 2,100 to one per 60,000 pregnancies, while it contributes to 1%-3% of ectopic pregnancies [1]. Ovarian pregnancy can be identified by the pathological criteria developed by Spiegelberg [2], which distinguish primary ovarian pregnancies from other ectopic pregnancies in which the ovary is secondarily involved; these criteria are as follows: (1) intact fallopian tube separate from the ovary on the involved side, (2) gestational sac in the normal position of the ovary, (3) the ovary with the gestational sac connected to the uterus by the ovarian ligament, and (4) the specimen having ovarian tissue attached to, and in, the wall of the gestational sac. Oftentimes, the presence of corpus luteum cysts and hemorrhagic cysts makes the sonographic diagnosis of an ovarian pregnancy difficult [3]. The factors associated with increased incidence of tubal ectopic pregnancies such as a history of pelvic inflammatory disease, previous ectopic pregnancy, history of infertility, or intrauterine contraceptive device do not seem to increase the risk of ovarian pregnancy [4]. Although the clinical sign and symptoms of ovarian pregnancy are similar to tubal ectopic pregnancies, sometimes, its vague symptoms produce bizarre clinical manifestations that can create a diagnostic illusion.

## Case presentation

A 29-year-old female patient with previous two cesarean deliveries visited the gynecology emergency department with the complaint of constant dull aching pain in the lower abdomen for seven days, which was aggravated by postural changes. She also complained of malaise, weakness, and breathlessness on brisk walking. She was alright 10 days prior at the onset of her menstrual periods, which was on scheduled time according to her menstrual history. She experienced severe dysmenorrhea for the first time as her previous cycles were never painful, for which she took some analgesics. The pain progressively worsened in severity, and she fainted. She was taken to a local hospital where she was evaluated and managed conservatively. She got her transabdominal sonography done, which showed no abnormality except for an echogenic lesion at the left renal cortex. She got discharged after her pain reduced, but there was constant dull aching pain in the lower abdomen since then. She also gave the history of using an intrauterine device for three years. Regarding her obstetric history, she had two live babies both delivered by cesarean section, and the age of the last child was three years at the time that she consulted our department.

On examination, the patient was severely pale, ill-looking, and anxious. Her pulse rate was 116 beats per minute, and her blood pressure was 96/60 mmHg. There was tenderness in the suprapubic area. Per vaginal examination, cervical motion tenderness was present, and uterine size could not be assessed due to significant tenderness.

The patient was admitted, and her transvaginal sonography was done along with a urine pregnancy kit test for the suspicion of ectopic gestation. The urine pregnancy test was positive. The blood report of the patient showed a low red blood cell count, and her hemoglobin was 7.1 g/dL. Transvaginal ultrasound showed an echogenic cyst-like structure in the left adnexa likely to be ruptured tubal or ovarian ectopic pregnancy, with collection in the pouch of Douglas and uterine cavity, with no evidence of intrauterine pregnancy.

**Figure 1:**
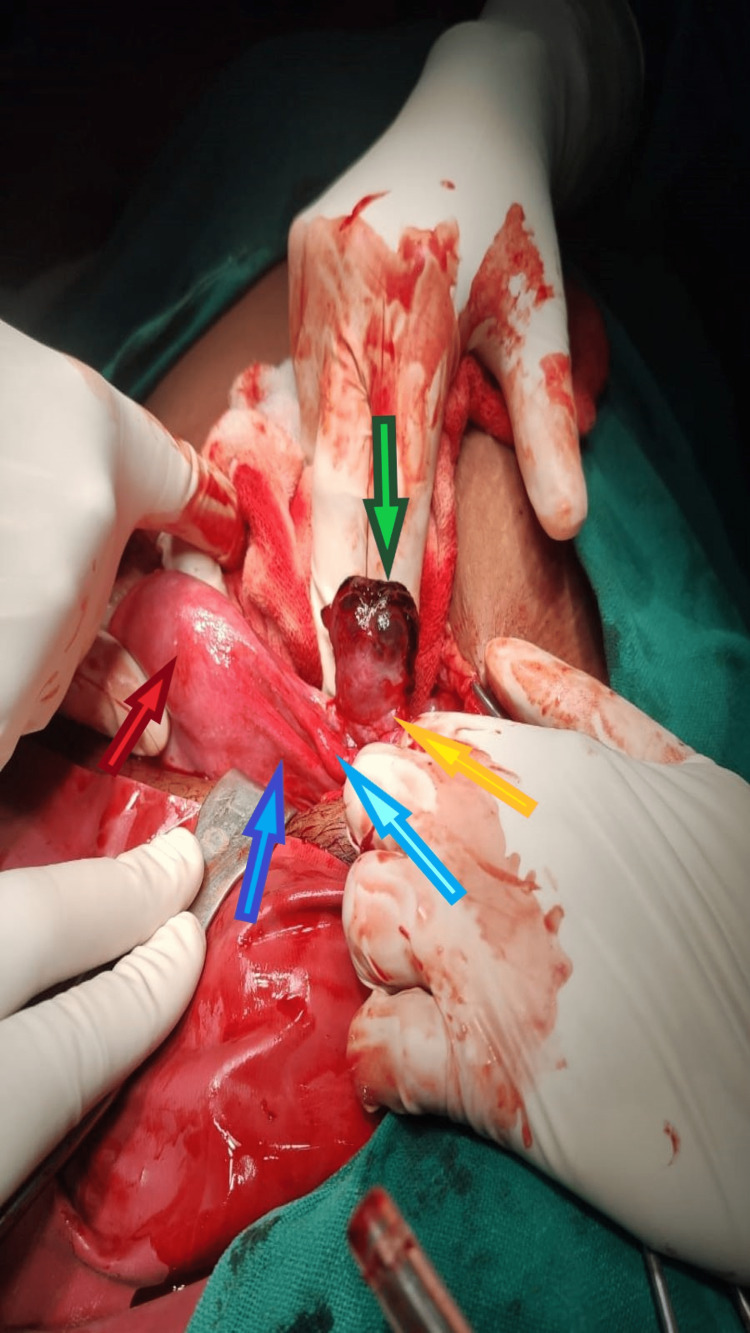
Ruptured ovarian pregnancy satisfying the Spiegelberg criteria. The green arrow shows an ovarian ruptured ectopic pregnancy, the yellow arrow shows the ovarian ligament, the sky blue arrow shows an intact healthy fallopian tube separate from the ovary, the violet arrow shows the round ligament, and the red arrow is showing the fundus of the uterus.

After the initial treatment and arrangement of two units of packed red blood cells after proper grouping and cross-matching for the patient, laparotomy was done. Clots were removed from the abdominal cavity. At the time of surgery, ruptured ovarian pregnancy on the left side was confirmed by the Spiegelberg criteria as shown in Figure 1. The patient elected for surgical contraception and underwent left salpingo-oophorectomy with right-sided tube ligation. The specimen was sent for histopathology examination, which confirmed the presence of a gestational sac attached to the ovarian tissue. The postoperative period of the patient was uneventful.

## Discussion

Ectopic pregnancies are extrauterine pregnancies, the majority of which occur in the fallopian tube with other possible but less involved sites such as cornual, intramural, cervical, cesarean scar, ovarian, or abdominal. Manifestations of tubal ectopic pregnancy appear typically at six to eight weeks after the last menses but may occur at a later duration in other sites of ectopic pregnancy. But in this case, the patient presented with the symptoms at four weeks of pregnancy and had a history of use of an intrauterine contraceptive device; these were confusing points and lead to the suspicion of tubal ectopic pregnancy, so focusing only on tubes for ectopic pregnancies during an ultrasound can mislead the diagnosis [5]. Transvaginal sonography is more reliable in detecting ectopic pregnancies in early gestation than transabdominal sonography [6]. Though it is challenging to distinguish between a hemorrhagic luteal cyst and an ectopic gestation, the degree of echogenicity of an adnexal mass can be of help [7]. The echogenicity of the corpus luteum is either equal to or less than the ovarian parenchyma, while the tubal ring of ectopic pregnancy is more echogenic. The onset of pain and vaginal bleeding was also confused with dysmenorrhea in this case, as its time of occurrence coincided with the scheduled time of menstrual periods. Fortunately, bleeding after the rupture of her ovarian pregnancy was not severe but organized, so she was hemodynamically stable until the presumptive diagnosis was made and surgery was performed. For the prevention of recurrence, patients should be informed that hormonal or intrauterine device reduces the risk of any pregnancy independent of the site of conception, but in the case of conception, then the chances of ectopic gestation are on the higher side than noncontraceptive users [8]. Patients should be advised to avoid smoking and vaginal douching as they may increase the risk of ectopic gestations.

## Conclusions

Bizarre and vague symptoms of an ovarian ectopic pregnancy can mislead clinicians and can be fatal for patients. This case report study suggests exploring all the possible sites of ectopic pregnancy during transvaginal sonography, even in the case of the slightest suspicion as timely intervention is very crucial for better prognosis of the patients.

Typically, the diagnosis of ovarian pregnancy is made at the time of surgery. To distinguish ovarian pregnancies from tubal pregnancies, strict histopathological criteria are used. Most of the time, treatment is done by laparotomy in hemodynamically unstable ones. With the advancement in the medical field and earlier detection of ectopic gestations, mortality due to ectopic pregnancy has reduced to a greater extent.
